# Improved fatty aldehyde and wax ester production by overexpression of fatty acyl-CoA reductases

**DOI:** 10.1186/s12934-018-0869-z

**Published:** 2018-02-08

**Authors:** Tapio Lehtinen, Elena Efimova, Suvi Santala, Ville Santala

**Affiliations:** Department of Chemistry and Bioengineering, Tampere University of Technology, Tampere, Finland

**Keywords:** Fatty acyl-CoA reductase, FAR, Fatty aldehyde, Wax ester, *Acinetobacter baylyi* ADP1

## Abstract

**Background:**

Fatty aldehydes are industrially relevant compounds, which also represent a common metabolic intermediate in the microbial synthesis of various oleochemicals, including alkanes, fatty alcohols and wax esters. The key enzymes in biological fatty aldehyde production are the fatty acyl-CoA/ACP reductases (FARs) which reduce the activated acyl molecules to fatty aldehydes. Due to the disparity of FARs, identification and in vivo characterization of reductases with different properties are needed for the construction of tailored synthetic pathways for the production of various compounds.

**Results:**

Fatty aldehyde production in *Acinetobacter baylyi* ADP1 was increased by the overexpression of three different FARs: a native *A. baylyi* FAR Acr1, a cyanobacterial Aar, and a putative, previously uncharacterized dehydrogenase (Ramo) from *Nevskia ramosa*. The fatty aldehyde production was followed in real-time inside the cells with a luminescence-based tool, and the highest aldehyde production was achieved with Aar. The fate of the overproduced fatty aldehydes was studied by measuring the production of wax esters by a native downstream pathway of *A. baylyi*, for which fatty aldehyde is a specific intermediate. The wax ester production was improved with the overexpression of Acr1 or Ramo compared to the wild type *A. baylyi* by more than two-fold, whereas the expression of Aar led to only subtle wax ester production. The overexpression of FARs did not affect the length of the acyl chains of the wax esters.

**Conclusions:**

The fatty aldehyde production, as well as the wax ester production of *A. baylyi,* was improved with the overexpression of a key enzyme in the pathway. The wax ester titer (0.45 g/l) achieved with the overexpression of Acr1 is the highest reported without hydrocarbon supplementation to the culture. The contrasting behavior of the different reductases highlight the significance of in vivo characterization of enzymes and emphasizes the possibilities provided by the diversity of FARs for pathway and product modulation.

**Electronic supplementary material:**

The online version of this article (10.1186/s12934-018-0869-z) contains supplementary material, which is available to authorized users.

## Background

Microbial synthesis of oleochemicals is an attractive option for the production of substitutes for the petrochemicals and fossil fuels [1, 2]. For the production of the long carbon chains required in oleochemicals, the fatty acid biosynthetic pathway is one of the few existing metabolic pathways [2]. Fatty acids and their activated forms (fatty acyl-CoAs and -ACPs) are precursors for a range of industrially relevant products, including alkanes, fatty aldehydes, fatty alcohols, triacylglycerols and wax esters [2]. Microbial production of these molecules has been achieved by the expression of native and/or heterologous enzymes in various host organisms [2, 3]. Although significant advancements in the understanding and manipulation of microbial lipid metabolism have been achieved, further consideration of the behavior and interaction of enzymes in different cell contexts is required in order to optimize the production and to diversify the range of possible products.

Of the bioproducts derived from acyl-CoA, aldehydes are of particular interest, as they represent industrially relevant molecules with a range of applications, from flavors and fragrances to precursors for pharmaceuticals [4]. The microbial production of volatile short-chain aldehydes has been improved by metabolic engineering [5]. In addition, aliphatic long-chain aldehydes are central intermediates in the biosynthesis of various industrially relevant lipid molecules, such as alkanes, fatty alcohols, and wax esters. Thus, the biosynthesis of these molecules would potentially benefit from increased long-chain aldehyde production. The key enzymes in aldehyde synthesis are fatty acyl-CoA (or -ACP) reductases (FAR). Various such reductases have been studied, including Aar from *Synechococcus elongatus* PCC 7942 [6], Aar-homologs from other cyanobacteria [7], and Acr1 from *Acinetobacter baylyi* ADP1 [8]. Notably, reductases found in marine bacterium *Marinobacter aquaeolei* VT8 [9, 10] or plants [11] further reduce the fatty aldehyde intermediate to fatty alcohol. Acr1 and Aar have been characterized not to reduce aldehydes [8, 12], and are thus more suitable for aldehyde production.

**Fig. 1:**
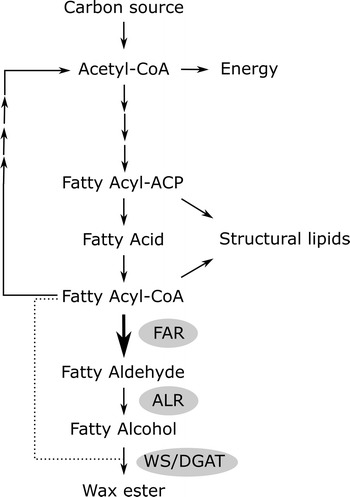
The outline of the proposed wax ester production pathway in *A. baylyi*. Wax esters are produced from fatty acyl-CoA in three steps (see text for details). In this study, the effect of the overexpression of different FARs on the pathway was studied. *CoA* coenzyme A, *ACP* acyl carrier protein, *FAR* fatty acyl-CoA reductase, *ALR* aldehyde reductase, *WS/DGAT* wax ester synthase/diacylglycerol acyl transferase

Depending on the cellular context and prevailing native or non-native enzyme activities, fatty aldehydes may have various fates in a cell, such as reduction to fatty alcohols, oxidation to fatty acids, or conversion to alkanes. For example, alkanes can be produced in a non-native microbial host by the expression of Aar and aldehyde-deformylating oxygenase (Ado), another enzyme originating from cyanobacteria [6, 13]. Aar catalyzes the reduction of acyl-ACP (or -CoA) to fatty aldehyde and Ado the conversion of the aldehyde to alkane [6]. In addition, the properties of the alkanes can be controlled by the selection of key enzymes with desired substrate specificities. Examples of this strategy include the expression of a modified thioesterase in *Escherichia coli* to modify the chain lengths of the alkanes produced with a synthetic pathway [14]. Another example of a pathway using fatty aldehyde as an intermediate compound is the synthesis of wax esters (WE), which are naturally produced by some bacterial species. The WE synthesis pathway in *Acinetobacter baylyi* ADP1 has been partially characterized in previous studies [8, 15]: the proposed pathway consists of three steps: (1) reduction of fatty acyl-CoA to fatty aldehyde by the fatty acyl-CoA reductase Acr1, (2) reduction of fatty aldehyde to fatty alcohol by a yet uncharacterized aldehyde reductase(s), and (3) esterification of fatty aldehyde with fatty acyl-CoA by a bifunctional wax ester synthase/diacyl glycerol acyl transferase (WS/DGAT) (Fig. 1).

In addition, *A. baylyi* ADP1 has been established as a robust chassis for synthetic biology, metabolic engineering, and genetic studies [13, 16–21]. It is particularly well suited for studying the fatty aldehyde and lipid production pathways, because of the tendency to naturally accumulate storage lipids. The acyl-CoA producing pathway is constantly active in favorable conditions [22], which simplifies the metabolic engineering process compared with other model organisms that require substantial modifications to the central carbon metabolism in order to promote efficient lipid synthesis [23]. In particular, the modularity of the WE pathway has been previously demonstrated by substituting the natural fatty acyl-CoA reductase Acr1 of *Acinetobacter baylyi* with a heterologous reductase complex LuxCDE in order to produce modified wax esters [24]. The genome of *A. baylyi* ADP1 has been sequenced and a metabolic model, as well as expression vectors and induction systems, are available [25–28]. Genetic engineering of *A. baylyi* ADP1 is straightforward due to the tendency for natural transformation and homologous recombination [20], enabling genome engineering in a high-throughput manner using automated systems [29].

As fatty aldehydes are rapidly metabolized in the cells, their analysis is often limited to indirect detection methods, such as conversion to alkanes [7] or alcohols [30]. We have previously developed a specific tool for monitoring the aldehyde production in the cells in real-time [13, 22, 31]. The tool is based on a bacterial luciferase LuxAB, which utilizes fatty aldehyde as a substrate in a reaction that produces visible light. Thus, aldehyde production can be directly detected in real-time during cell growth, offering a convenient tool for screening and optimization of the aldehyde-producing enzymes. In this study, we utilized the previously developed real-time aldehyde monitoring tool and the natural WE production machinery of *A. baylyi* ADP1 to study the fatty aldehyde overproduction and to elucidate the fate of the overproduced fatty aldehydes in the cells. In addition to the previously characterized FAR enzymes Acr1 and Aar, an uncharacterized putative dehydrogenase from a bacterium *Nevskia ramosa* was included in the study.

## Methods

### Bacterial strains and genetic modifications

The bacterial strains used in the study are listed in Table 1. The wild type *A. baylyi* ADP1 (DSM 24193, Deutsche Sammlung von Microorganismen und Zellkulturen, Germany) was used in the study. For cloning and plasmid amplification, *E. coli* XL1-Blue (Stratagene, USA) was used. The molecular work was carried out using established methods. The reagents and primers were purchased from ThermoFisher Scientific (USA) and used according to the manufacturer’s instructions. Transformation of *A. baylyi* was carried out as described earlier [32]. Antibiotics were used for the selection with concentrations of 25 µg/ml for chloramphenicol and 50 µg/ml for kanamycin and spectinomycin. All genetic modifications were confirmed with PCR and sequencing.

**Table 1:** Bacterial strains used in the study

Name	Genotype	Description
S1	ADP1Δ*ACIAD3381*–*3383*::*luxAB*,*kan*^*r*^	Background strain for FAR overexpression with LuxAB for luminescence measurements (native FAR deleted)
iluxAB	ADP1Δ*ACIAD3381*::*luxAB*,*cm*^*r*^	Control strain for S1 (with LuxAB), carrying native FAR (Acr1)
W1	ADP1Δ*ACIAD3381*–*3383*::*cm*^*r*^	Background strain for FAR overexpression without LuxAB
ADP1Δ3381	ADP1Δ*ACIAD3381*::*kan*^*r*^	Control strain for W1 (without LuxAB), carrying native FAR (Acr1)

ACIAD3381—*poxB*; ACIAD3383—*acr1*

For construction of the background strains (S1 and W1), a previously described gene cassette was used [32]. Briefly, the cassette contains chloramphenicol resistance marker and homologous regions to facilitate genomic integration. The cassette is designed to knock out the ACIAD3383–3381 genes, thereby removing the endogenous fatty acyl-CoA reductase Acr1 and pyruvate oxidase PoxB activities. For S1, the kanamycin resistance marker was used, and for W1 the marker was chloramphenicol resistance [32]. For the S1 strain, the *luxAB* genes were cut from the *iluxAB_Cm*^*r*^/pAK400c plasmid [22] using *Nde*I and *Xho*I restriction enzymes and cloned to the cassette under T5/*lac* promoter. ADP1 was transformed with either of the constructs and selected with the respective antibiotic.

For FAR overexpression, a construct was prepared where the corresponding FAR gene is under T5/*lac* promoter in an integrative cassette. The integrative region of the plasmid pIM1463 [25] was PCR amplified in two parts with primers tl24, tl25, tl26 and tl27 (Additional file 1: Table S2) and designed in such a way that the *gusA* gene becomes replaced by a Biobrick RFC10 accepting cloning site (*Eco*RI-*Xba*I-*Spe*I-*Pst*I). The T5/*lac* promoter and the *lacI* gene were preserved as they are in the original plasmid. The construct contained flanking sites to facilitate the integration into a prophage region in the genome of *A. baylyi* ADP1. *Acr1* (ACIAD3383) was amplified from the genome of *A. baylyi* ADP1 and *ramo* (an open reading frame encoding for a putative short chain dehydrogenase WP_022976613.1) from the genome of *Nevskia ramosa* (DSM 11499) with primers tl17 and tl18 and tl33 and tl34, respectively (Additional file 1: Table S2). *Aar* (*S. elongatus PCC7942_orf 1594*) was codon-optimized and purchased from Genscript (USA) with appropriate restriction sites and ribosomal binding site. The three genes were separately cloned to the Biobrick cloning site of the constructed cassette. The nucleotide sequences of the three genes, as well as the sequence and plasmid map of the expression cassette, are provided in the Additional file 1.

A control strain for the S1 strain was a previously described strain *A. baylyi* ADP1∆*poxB*::i*luxAB_Cm*^*r*^ [22], in which the gene ACIAD3381 is replaced with a cassette containing the *luxA* and *luxB* genes. A control strain for the W1 strain was *A. baylyi* ADP1Δ*poxB*::*Kan*^*r*^/*tdk* [32], where the gene ACIAD3381 is replaced with a kanamycin resistance marker [29]. Both control strains were transformed with the empty expression cassette (expressing only *lacI* under the T5/*lac* promoter).

### Medium and culture conditions

A modified minimal salts medium MA/9 was used for the cultivations. The medium contained (per liter): 5.52 g Na_2_HPO_4_·2H_2_O, 3.4 g KH_2_PO_4_, 1 g NH_4_Cl, 0.008 g nitrilotriacetic acid, 0.5 mg FeCl_3_, 11.1 mg CaCl_2_ and 240 mg MgSO_4_. The medium was supplemented with 0.2% w/v casamino acids and 5% d-glucose. IPTG (isopropyl β-d-1-thiogalactopyranoside) was used for induction in concentrations indicated in the results section. For the luminescent measurements, the cells were incubated inside Xenogen In Vitro Imaging System (IVIS^®^ Lumina, Caliper Life Sciences, USA) in liquid culture on 48-well plate (1 ml medium/well) at 30 °C or room temperature, and without shaking. The total flux of photons from each well was measured every hour with a 10-min exposure time. For WE production experiments, the cells were cultivated in tubes (5 ml volume) or flasks (50 ml volume) for 48 h with 300 rpm shaking and temperature as indicated in the result section. The optimal temperature for A. baylyi growth is 30 °C, and the growth is slower at lower temperatures. The cultivation time (48 h) is long enough to ensure that all the cultures have reached stationary phase at the time of WE analysis.

### Lipid extraction and analysis

Small-scale lipid extraction for the thin layer chromatography (TLC) analysis was carried out as follows. Cells from equal volumes (1–3 ml) of the cultures were collected by centrifugation and suspended in 500 µl of methanol. Next, 250 µl of chloroform was added and samples were incubated with shaking for 1 h. Then 250 µl of chloroform and 250 µl of PBS added and incubated for 1 h with gentle mixing. The samples were centrifuged and the lowest phase used for TLC analysis. The TLC analysis was carried out using 10 × 10 cm Silica Gel 60 F254 HPTLC glass plates with 2.5 × 10 cm concentrating zone (Merck, USA). Mobile phase was *n*-hexane: diethyl ether: acetic acid, with proportions 90:15:1, respectively. Iodine was used for visualization. Jojoba oil was used as an external wax ester standard.

For the fatty aldehyde and fatty alcohol analysis by gas chromatography-mass spectrometry (GC–MS), cells from 10 ml of the cultures were collected and the lipids extracted as described above. Additionally, the lipids from the supernatant were extracted by mixing 1 ml of the supernatant and 1 ml of ethyl acetate vigorously for 10 min. The lower phase (chloroform) from the biomass extraction and the upper phase (ethyl acetate) from the supernatant extraction were applied to the GC–MS analysis. GC–MS analysis was performed with Agilent Technologies 6890N/5975B system, column HP-5MS 30 m × 0.25 mm with 0.25 µm film thickness, He flow 4.7 ml/min, 1 µl splitless injection, oven program: 55 °C hold 5 min, 55–280 °C 20°/min ramp, 280 °C hold 3 min. Scan 50–500 *m/z*, 1.68 scan/s. Identification of the peaks was based on the NIST library (Version 2.2/Jun 2014) and external standards (1-hexadecanol and 1-octadecanol, Sigma-Aldrich, USA).

For the wax ester analysis with NMR and GC, the cells from 40 ml of the cultures were collected by centrifugation (30,000*g*, 30 min) and the cell pellets were freeze-dried with ALPHA 1–4 LD plus freeze-dryer (Martin Christ, Germany). The extraction of lipids from freeze-dried biomass and the quantitative ^1^H NMR analysis of wax esters was carried out as described earlier [22]. The areas of the peaks in the NMR spectrum are directly proportional to the molar concentration of each functional group. The content of wax esters in total lipid extracts was calculated from the integrated signal at 4.05 ppm which is characteristic for protons of α-alkoxy-methylene group of esters (–CH_2_-COO-CH_2_–). For the calculation of the WE titer in grams per liter, an average molar mass of 506 g/mol was used (corresponding to a WE with 34 carbons and one double bond).

For the analysis of the wax ester composition, the wax esters were purified from the lipid extract and analyzed by NMR and gas chromatography (GC). Purification and isolation of wax esters were performed by preparative TLC, employing aluminum sheets precoated with silica gel 60 F254 (Merck) and *n*-hexane: dichloromethane 3:2 as mobile phase. The products with R_f_ 0.42 were scraped from TLC plates and extracted from silica gel with chloroform with 5% of ethanol. The solutions were filtered from silica gel and solvents were removed in vacuum. The isolated wax ester fractions were analyzed by ^1^H NMR spectroscopy. The average degree of unsaturation and the average length of fatty chains were calculated from NMR spectra as described earlier [33]. For GC, the isolated wax esters were hydrolyzed and transmethylated and peaks were qualitatively identified based on external standards. See Additional file 1 for details about NMR and GC analyses.

## Results

We selected two previously characterized and one putative reductase from different organisms and studied the effect of their expression on aldehyde production in *A. baylyi* ADP1. The first one was the endogenous fatty acyl-CoA reductase Acr1 from *A. baylyi*. In addition, Aar from *S. elongatus* (PCC7942_orf1594) and an Acr1-homolog (WP_022976613.1, here designated Ramo) from *N. ramosa* were studied. Aar is characterized to accept both acyl-ACP (acyl carrier protein) and acyl-CoA (coenzyme A) as substrates [6], but Acr1 only accepts acyl-CoA [8]. Ramo has not been previously studied and thus the function as a reductase was not known. The gene was identified with a BLAST search based on its homology to *acr1*. A preliminary in vivo characterization of Ramo in *E. coli* indicated fatty aldehyde production associated with its expression (Additional file 1).

To generate a background strain for the study, the wild type ADP1 was transformed with a genetic cassette containing the *luxA* and *luxB* genes and a kanamycin resistance marker. The construct also contained homologous flanking regions facilitating its integration to the genome and deletion of the genes ACIAD3381–3383 by homologous recombination. The gene ACIAD3383 encodes the natural fatty acyl-CoA reductase Acr1, and thus the natural aldehyde production related to the wax ester production pathway was removed [8, 22]. The gene ACIAD3381 encodes a pyruvate oxidase, and the deletion of this gene has been associated with higher WE production in an earlier study [32]. In addition to these two genes, the ACIAD3382 gene was deleted for practical reasons to allow the deletion of ACIAD3383 and ACIAD3381 with a single cassette. ACIAD3382 encodes for a homocysteine synthase, and its deletion has been shown to be neutral in terms of growth and lipid production [32]. The strain used in this study has a genotype ADP1Δ3381–3383::luxAB,kan^r^ and was designated S1. A control strain was the previously described *A. baylyi* ADP1∆*poxB*::i*luxAB_Cm*^*r*^, designated here as iluxAB strain [22], in which only ACIAD3381 is replaced by the luxAB-containing cassette. This strain has the wild type expression of Acr1, and therefore can be used as a control for studying the effect of the overexpression.

**Fig. 2:**
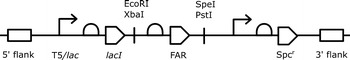
The gene cassette used for the overexpression of the FARs. The cassette contains the *lacI* and FAR genes under T5/*lac* promoter, a spectinomycin resistance marker under its own promoter and homologous flanking sites facilitating the integration of the cassette to the prophage region of *A. baylyi* genome

The three different FARs were separately cloned under T5/lac promoter in a cassette containing the *lacI* gene, a spectinomycin resistance marker, and homologous sequences facilitating its integration to a prophage region of ADP1 genome [25] (Fig. 2). The constructed cassettes were introduced to the S1 strain.

**Fig. 3:**
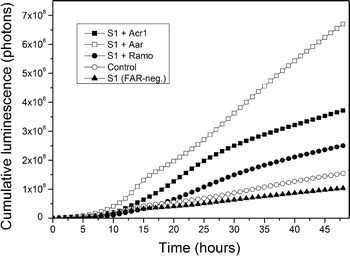
The effect of FAR overexpression on aldehyde production. All the strains express the luciferase LuxAB, which reacts with fatty aldehydes to produce the measured luminescence signal. The S1 strain expressing Acr1, Aar or Ramo under an IPTG-inducible promoter were cultivated at 30 °C for 48 h and the luminescence signals measured. The control strains were iluxAB, which has a wild type expression of Acr1 and S1, which is FAR negative. Various IPTG concentrations were tested and the ones that yielded the highest signal are presented here. The concentrations were 1 mM for Acr1 and Aar, and 0.1 mM for Ramo. The full data with all the tested IPTG concentrations is presented in Additional file 1: Figures S2, S3. The signals are presented as averages of two independent replicates. Standard deviations were negligible and thus omitted for clarity

Our previously developed bioluminescence-based tool enables the monitoring of the aldehyde production in the cells in real-time [13, 22, 31]. The aldehyde production can be directly detected during cell growth. Thus, it was expected that increasing the expression of FARs would increase the long chain aldehyde production, which would be seen as an increase in the luminescent signal. The cells were grown at 30 °C, the expression of the FARs was induced with different concentrations of IPTG and the luminescence response monitored in real time as the cells grew (Fig. 3). For each of the three genes, the luminescence signal could be detected, indicating that the reductases were functionally expressed and provided aldehydes as suitable substrates for LuxAB. Moreover, the luminescence production associated with the expression of the putative dehydrogenase Ramo suggests that its expression leads to fatty aldehyde production in this cellular context. With all the genes, the luminescence signals increased with increasing IPTG concentrations. The signals were also markedly higher than the signal from the control strain iluxAB, indicating increased internal aldehyde production (Fig. 3). The IPTG concentration did not have a significant effect on the luminescence of the iluxAB strain (Additional file 1: Figure S2) confirming that changes in the expression levels of *luxA* and *luxB* are not limiting the luminescence production, as has been shown earlier [22]. The highest tested concentration of 1 mM IPTG yielded the highest luminescence response for Aar and Acr1, whereas for Ramo the highest luminescence signal was obtained with 100 µM IPTG (Additional file 1: Figure S3). With Ramo, the signal obtained with 1 mM IPTG was slightly lower than with 100 µM, possibly due to the increased metabolic burden associated with the high expression level. These data demonstrate that the aldehyde production in *A. baylyi* can be increased in a controllable manner with the overexpression of any of the three enzymes tested.

In order to study the effect of the temperature on the luminescence production, the experiment was performed also at room temperature (ca. 24 °C). At this temperature, the luminescence signals from the overexpression strains were close to the wild type strain (Additional file 1: Figure S4). The effect of the increasing IPTG induction on the luminescence signal was similar as at 30 °C (i.e. the highest signals were obtained with the highest IPTG concentration (1 mM) with Acr1 and Aar, whereas with Ramo the highest signal was obtained with 100 µM IPTG).

Fatty aldehydes are usually rapidly metabolized by the cells [34, 35]. Thus, the fate of the overproduced fatty aldehydes in the cells was studied. For this purpose we utilized the natural wax ester production pathway present in *A. baylyi* [8, 15], for which fatty aldehyde is a specific intermediate. Based on the luminescence results, we hypothesized that at least part of the overproduced fatty aldehydes would be directed to wax ester synthesis pathway and thus lead to increased WE production.

**Fig. 4:**
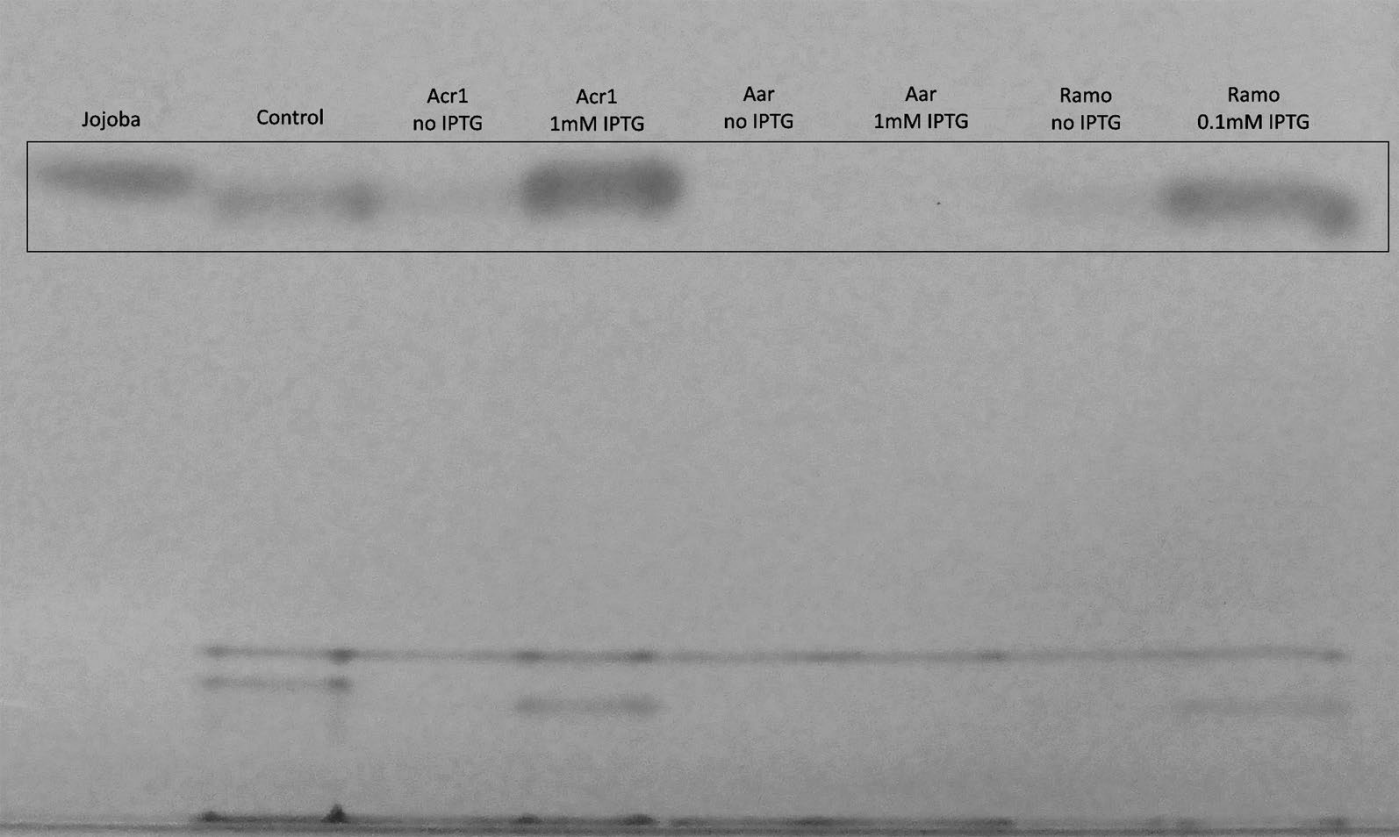
Thin layer chromatography analysis demonstrating the effect of the FAR overexpression on the WE production. The S1 strain harboring different FARs under an IPTG-inducible promoter were cultivated at 30 °C for 48 h with or without IPTG, the lipids extracted and analyzed with thin layer chromatography. The control strain iluxAB has the wild type expression of Acr1. Jojoba oil (1 mg/ml) was used as a wax ester standard. Equal volume of each culture were used for the extraction. Two replicates were analyzed and a representative picture is shown. The clarity of the picture was enhanced by adjusting the contrast

The S1 strains overexpressing Aar, Ramo, or Acr1 were cultivated at 30 °C with the IPTG concentration that led to the highest luminescence production, and WEs were analyzed with thin layer chromatography. It was found that the overexpression of Acr1 or Ramo resulted in markedly higher WE production compared with the control cells with wild type Acr1 expression (Fig. 4). On the other hand, the Aar-expressing strain produced only a small amount of WEs, despite the higher luminescence signal. The amount of WEs produced by the Aar-expressing strain was under the detection limit of TLC (Fig. 4), but the production was confirmed with NMR analysis (17 mg/g CDW, 60 mg/l). Thus, intriguingly, even though the aldehydes produced by the different enzymes can be detected with the LuxAB-based sensor, they seem to be diverted to different downstream pathways. The FAR-negative background strain did not produce wax esters, as expected.

Wax ester production in *Acinetobacter* is strongly affected by temperature, with higher production in low temperatures [24]. Thus, we wanted to study the effect of the overexpression of FAR (endogenous or heterologous) on the WE production in different temperatures. We hypothesized that the overexpression of a key enzyme in the pathway could at least partly unravel the temperature dependency of the WE production. Because the aldehydes produced by Aar were found not to be directed to WEs in the cells, it was excluded from this experiment. In this point, the background strain was changed from the *luxAB*-containing S1 to W1. The strain W1 is otherwise identical to S1 but lacks the *luxAB* genes and has a chloramphenicol resistance marker instead of the kanamycin marker (ADP1ΔACIAD3381–3383::cm^r^).

W1 strains harboring either *acr1* or *ramo* were cultivated at 20, 30 and 37 °C for 48 h. The inducer concentrations were chosen according to the highest luminescence signals obtained in the previous experiment; 1 mM for Acr1 and 0.1 mM for Ramo. According to quantitative NMR analyses, the overexpression of the reductases at 30 °C resulted in more than two-fold increase in the WE content of the cells for both Acr1 and Ramo compared with the control strain (Table 2). On the contrary, the overexpression at 20 °C did not significantly improve the production in comparison with the control strain (Table 2). At 37 °C, the overexpression did not improve the WE production (based on TLC analyses). The increase in the WE production was accompanied by only a small decrease in cell growth. Thus, the titer, productivity, and yield were also enhanced by approximately twofold at 30 °C (Table 2). The WE titer (0.45 g/l), productivity (9.4 mg/(l h)) and yield (0.040 g/g glucose) were highest for the Acr1 overexpressing strain grown at 20 °C, but the overexpression of the FARs enhances the WE production at 30 °C to a level comparable to that of at 20 °C (Table 2). Thus, the aldehyde overproduction effectively decouples the temperature dependency of WE production, enabling high production at the optimal growth temperature (30 °C) of *A. baylyi*.

**Table 2:** The effect of the FAR overexpression on the WE production in different temperatures

T (°C)	Strain	CDW (g/l)	WE content^a^ (mg/g CDW)	WE titer^a^ (g/l)	Productivity^a^ (mg/(l h)	Yield^a^ (g/g glucose)
30	Control	5.6 ± 0.2	35 ± 2	0.20 ± 0.01	4.1 ± 0.3	0.014 ± 0.001
	W1 + Acr1	4.3 ± 0.5	93 ± 3	0.40 ± 0.06	8.4 ± 1.3	0.032 ± 0.003
	W1 + Ramo	5.3 ± 0.3	78 ± 8	0.41 ± 0.07	8.6 ± 1.4	0.025 ± 0.005
20	Control	4.0 ± 0.06	110 ± 0	0.43 ± 0.01	9.0 ± 0.2	0.035 ± 0.002
	W1 + Acr1	3.7 ± 0.02	125 ± 11	0.45 ± 0.04	9.4 ± 0.8	0.040 ± 0.002
	W1 + Ramo	4.1 ± 0.08	109 ± 5	0.45 ± 0.03	9.4 ± 0.6	0.040 ± 0.007

The W1 (ADP1Δ3381–3383::cm^r^) cells expressing either Acr1 or Ramo under an IPTG-inducible promoter were cultivated at 30 and 20 °C for 48 h, the lipids extracted and wax esters measured with NMR. The average and standard deviation of two independent cultivations are shown. The control strain was ADP1Δ3381, which has the wild type Acr1 expression

*CDW* cell dry weight, *WE* wax ester

^a^An average molar mass of 506 g/mol for wax esters used in the calculations

The physical and chemical properties of WEs are largely determined by the length of the carbon chain and the level of unsaturation. In *Acinetobacter*, the wax esters typically consist of carboxyl and alcohol moieties with 16 or 18 carbons, the total length of the carbon chain being 32–36 [36]. The fatty acyl chain length of WEs is probably largely determined in the point of termination of the fatty acyl chain elongation. However, it is possible that FARs affect the composition of WEs for example through a biased substrate specificity [24]. In order to determine whether the overexpression of Acr1 or Ramo has an effect on the carbon chain length, we studied the composition of the WEs with NMR and gas chromatography. The cells were cultivated at 30 °C for 48 h and the WEs extracted, purified and analyzed. The composition of WEs was found to be similar to those of the wild type, with approximately 34 carbons and one double bond in a wax ester molecule (Additional file 1).

Because the fatty aldehydes produced by Aar were not efficiently directed to WE synthesis, we investigated alternative fates for the aldehydes. Even though aldehydes inside the cells are usually rapidly metabolized, fatty aldehydes could potentially be secreted out from the cells and thus accumulate in the culture medium. Another possibility is the conversion of fatty aldehydes to fatty alcohols by endogenous enzymes [7, 34]. The W1 cells overexpressing Aar or Acr1 were cultivated at 30 °C with 1 mM IPTG induction, and fatty aldehydes and fatty alcohols were analyzed from both the biomass and the supernatant by GC–MS. The fatty aldehyde concentration was below the detection limit in all samples both in the supernatant and in the biomass. Fatty alcohols were detected from the biomass samples but not from the supernatant. The fatty alcohol concentration was decreased in the Aar-overexpressing strain compared to the Acr1-strain and the wild type (Additional file 1: Table S1).

## Discussion

Fatty acyl-CoA/ACP reductases are key enzymes in the production of fatty acyl-derived molecules, and earlier studies have indicated that this step is limiting the production of downstream products, such as fatty alcohols, in synthetic pathways [37, 38]. Thus, the identification and in vivo characterization of modular and orthogonal reductases that can be utilized in diverse metabolic engineering strategies is of high importance. In this study, we studied the effect of the overexpression of three different FARs in *A. baylyi* ADP1. Two well-studied aldehyde-producing (as opposed to alcohol-producing) FARs were selected for the study (Aar and Acr1). In addition, in an attempt to broaden the array of available reductases, an uncharacterized putative dehydrogenase from *Nevskia ramosa* was included in the study.

First, the effect of FAR overexpression on aldehyde production was analyzed with a bioluminescence-based sensor. It was shown that the aldehyde production in *A. baylyi* can be increased and controlled by regulating the expression any of the three studied enzymes. The highest luminescence signal was obtained with Aar. In an earlier study [30], Acr1 was more efficient in aldehyde production than Aar in *E. coli*. The difference can be due to the different detection method; Liu et al. converted the aldehydes to alcohols before analysis whereas we employed a direct luciferase-based method for the detection of aldehydes, further emphasizing the importance of monitoring the target metabolite of interest in determining the enzyme activity.

The metabolic fate of the overproduced fatty aldehydes was studied by utilizing the natural wax ester production pathway. It was shown that the increased aldehyde production correlated with increased wax ester production with Acr1 and Ramo. Thus, the aldehyde overproduction is reflected also in the downstream production efficiency. The overexpression did not impose a large burden to the cells, as demonstrated by only a small degrease in cell growth. Consequently, the increase in the WE content of the cells was reflected as an increase in also titer, productivity, and yield.

In contrast to Acr1 and Ramo, the fatty aldehydes produced by Aar were not efficiently directed towards the WE production. The reasons for this are not clear, but it can be speculated that the subcellular localization of the enzymes affect the flux of the metabolites through the WE synthesis pathway. Acr1 is speculated to be a membrane-associated protein [8], whereas Aar is soluble [12]. WS/DGAT has also been observed to be associated with the membranes [39], and it is therefore plausible to assume the whole wax ester synthesis pathway to be spatially closely arranged in the cells, ensuring efficient delivery of the intermediates between the enzymes. Since Aar is a soluble enzyme, the aldehydes produced by Aar might not be efficiently directed to these downstream enzymes. Another possible reason could be the fact that Aar also accepts acyl-ACPs as substrates, which could affect the balance of the activated acyl molecules in the cell, negatively affecting the wax ester production.

Fatty aldehydes are usually efficiently converted to the corresponding alcohols by endonous aldehyde reductases in the cells [6, 34]. However, the Aar-overexpressing strain accumulated less fatty alcohols than the wild type or the Acr1-overexpressing strain, indicating that the flux from Aar to the yet uncharacterized endogenous fatty aldehyde reductase(s) of *A. baylyi* is inefficient. As the aldehyde reduction to alcohol is the next step in the WE production pathway (Fig. 1), this indicates that the aldehyde reductase is responsible for the inefficient WE production of the Aar-overexpressing strain.

It is also possible that the subcellular distribution of fatty aldehydes affects the signal obtained with the LuxAB biosensor. The luminescent signal is expected to reflect the concentration of fatty aldehydes available for the luciferase enzyme. The aldehydes produced by Aar might be more readily available for the luciferase, which could partially explain the high luminescent signals obtained. If this is the case, Aar could potentially be an optimal reductase for the production of other aldehyde-derived products, such as alkanes. We have previously produced alkanes in the *A. baylyi* host by the expression of the two-step cyanobacterial pathway involving Aar and Ado (aldehyde deformylating oxygenase) [13]. In this pathway, Aar produces fatty aldehydes, which are converted to alkanes by Ado. Thus, the successful implementation of this pathway in *A. baylyi* indicates that the aldehydes produced by Aar are readily available for other enzymes in the cell, even though they do not support efficient wax ester synthesis.

Interestingly, while aldehydes produced by Aar were not directed to WE synthesis, the expression of another heterologous enzyme (Ramo) promoted efficient WE synthesis. The performance of Ramo was comparable to that of the endogenous Acr1, demonstrating that it is possible to improve the WE production by the expression of a heterologous enzyme. To our knowledge, this is the first report describing the heterologous expression of Ramo (WP_022976613.1) from *Nevskia ramosa*. The protein is annotated as a short chain dehydrogenase based on sequence, but little is known about the enzyme’s substrate, end product, or other characteristics. In this study, we observed a correlation with fatty aldehydes and the expression of the *ramo*, indicating that fatty aldehyde is at least one of the end products in the cellular context of ADP1. Further studies would need to be carried in order to determine the details of the substrate specificity and other characteristics of the enzyme. Various reductases are widely used in metabolic engineering, and the results presented here suggest Ramo to be a useful part in the toolbox.

The contrasting behavior of Aar and Ramo underlines that even enzymes of seemingly similar function need to be studied in the context of the production chassis. In addition, an enzyme’s activity is not necessarily reflected in the quantity of the final product of the pathway. Thus, to avoid futile use of cellular resources and substrates, biological sensors that allow the detection of intermediate metabolites in real time are valuable tools in determining the in vivo activity and efficiency of heterologous enzymes. On the other hand, the differential behavior of the FARs provide possibilities to construct pathways with diverse products.

WE synthesis in *A. baylyi* has been previously observed to be more efficient at lower temperatures [24]. At the optimal growth temperature (30 °C), carbon is efficiently directed to biomass formation and not WE synthesis. With lower temperatures, the growth is slower but a larger proportion of carbon is directed to wax esters [24]. This may be due to the general tendency of *A. baylyi* to produce more WEs at lower growth rates [36], or for example adaptation to the colder environment. Based on the data presented here, FAR overexpression effectively decouples this temperature dependency and enables simultaneous high WE production and high growth rate at 30 °C. This demonstrates the natural regulatory mechanisms for WE accumulation can be overridden by the expression of a key enzyme in the pathway.

The storage lipid accumulation in various microorganisms is promoted by an excess of available carbon source [36, 40, 41]. Thus, a common strategy to improve the lipid production is to perform a two-phase cultivation, where biomass is first accumulated in an optimal growth medium and in the second phase, a medium with high carbon-to-nitrogen ratio is used to promote efficient lipid synthesis. In this work, we studied the effect of FAR overexpression in the aldehyde formation as well as the quality and quantity of WEs in a simple one-phase cultivation without optimization of the carbon to nitrogen ratio. Such optimization combined to the FAR overexpression could potentially further increase the WE titer. Even without this optimization, the WE titer (0.45 g/l) and productivity (9.4 mg/(l h)) achieved in this study are higher than previously reported with microbial production systems involving de novo synthesis of the fatty acyl chains (i.e. without hydrocarbon supplementation to the cultivation).

## Conclusions

In this work, the fatty aldehyde production in *A. baylyi* was increased by the overexpression of any of the three fatty acyl-CoA reductases. With two of the reductases (Acr1 and Ramo), the increased aldehyde production was associated with an increase of the downstream product, wax esters. The wax ester titer and productivity reported here are the highest among the literature without hydrocarbon supplementation to the cultivation. With the third reductase (Aar), the overproduced fatty aldehydes were not directed to the WE synthesis, highlighting the importance of in vivo characterization of heterologous enzymes.

## Additional file

**Additional file 1.** Supplementary data and methods.
