# Supplementary material for: Improved fatty aldehyde and wax ester production by overexpression of fatty acyl-CoA reductases
Source: Microb Cell Fact. 2018 Feb 8;17:19. doi: 10.1186/s12934-018-0869-z (PMC5806253; doi:10.1186/s12934-018-0869-z)
Supplement: Supplementary file 1 — Additional file 1. Supplementary data and methods. [file 12934_2018_869_MOESM1_ESM.docx]

**Additional File 1**

Improved Fatty Aldehyde and Wax Ester Production by Overexpression of Fatty Acyl-CoA Reductases

Tapio Lehtinen, Elena Efimova, Suvi Santala, Ville Santala

**Initial characterization of Ramo**

In order to identify novel candidate genes for the fatty aldehyde production, homologs for *A. baylyi* Acr1 were searched with BLAST from the non-redundant protein sequence database. Among the top results, a putative short chain hydrogenase (WP_022976613, designated here as Ramo) from *Nevskia ramosa* was selected for further studies. *Nevskia Ramosa* is an aerobic bacterium specialized in living in the air-water interface. The selection of this homolog was supported by 1) The fact that the whole genome sequence for *N. ramosa* was available also in the beginning of this study and 2) the genomic sequence revealed the presence of a WS/DGAT homolog, indicating a possible wax ester production pathway, and thus supporting the possibility that WP_022976613 could be involved in fatty aldehyde metabolism.

The *ramo* gene was PCR amplified from *N. ramosa* genome and cloned into expression vector pMIK30 under T5/*lac* promoter. The aldehyde-producing activity of Ramo was characterized *in vivo* in *Escherichia coli*. *E. coli* KRX cells harboring a luxAB/pIX plasmid (ColE1 origin of replication) were transformed with the *ramo*/pMIK30 plasmid (p15A origin of replication). The bacterial luciferase luxAB catalyzes a reaction converting fatty aldehyde to fatty acid and visible light. Thus, the aldehyde production in the cells can be monitored by following the luminescence. The cells were cultivated overnight and diluted to optical density of 0.5. The expression of Ramo was induced with IPTG, cells grown for 4 hours, and the aldehyde production measured using the LuxAB system. An increasing luminescent signal was observed with increasing IPTG concentration (Figure S1), indicating that the expression of *ramo* leads to fatty aldehyde production *in vivo*.


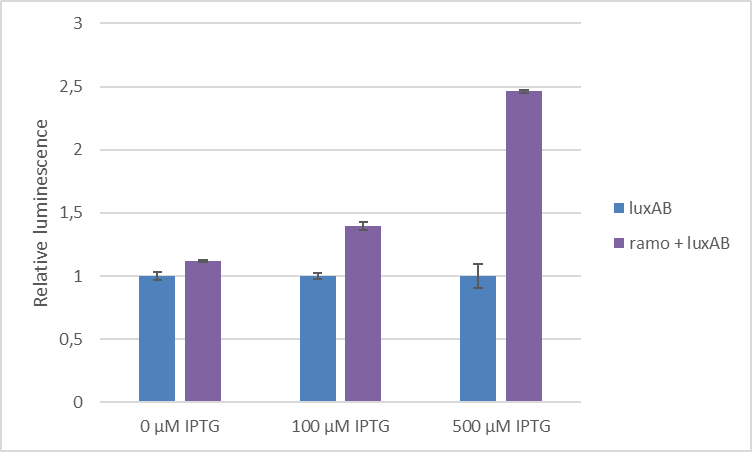


**Figure S1.** *In vivo* measurement of aldehyde production by Ramo. *E. coli* KRX cells harboring the *ramo* gene under an IPTG-inducible promoter and the *lux A* and *lux B* genes were cultivated with different IPTG concentrations and the luminescence signal measured. The fold change of the luminescence signal relative to the control strain harboring only the *lux A* and *B* genes is presented.

**Luminescent measurements**

The luminescent data complementing the data in Figure 3 are presented in Figures S2 and S3. The luminescent data from a similar experiment performed at lower temperature (room temperature, ca. 24°C) is presented in Figure S4.

**

**

**Figure S2.** The luminescence production with the wild type Acr1 expression. The iluxAB cells harboring the empty expression cassette (with *lacI*, but not the FAR genes) were cultivated for 48 hours at 30°C and the luminescence signals measured. This strain has the wild type Acr1 expression, and thus the aldehyde production is expected to be independent of IPTG concentration. The data is from the same experiment as Figure 3 and Figure S3. The averages of two replicates are shown.





**Figure S3.** Luminescence production with different expression levels of the three genes. The S1 strain expressing Acr1, Aar or Ramo under an IPTG-inducible promoter were cultivated with varying concentrations of IPTG at 30°C for 48 hours and the luminescence signals measured. The data is from the same experiment, but is presented in separate graphs for clarity. The control strains iluxAB (with wild type Acr1 expression) and S1 (FAR-negative strain) are shown in each graph as a reference. The averages of two replicates are shown.


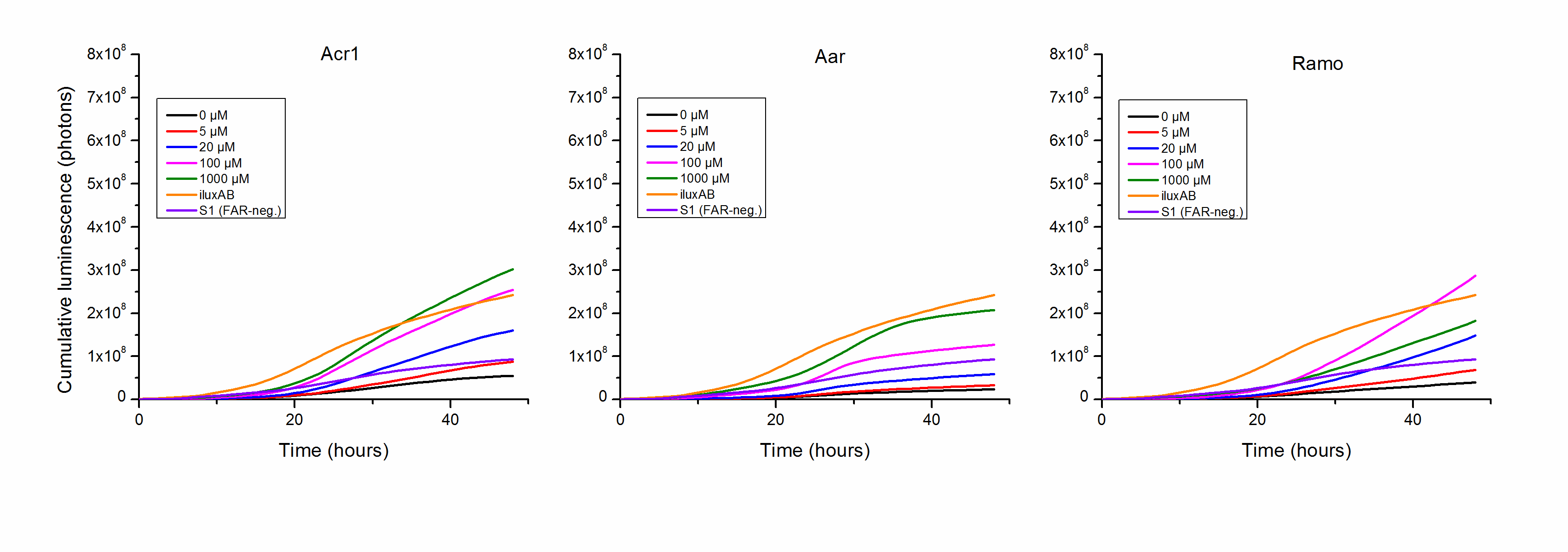


**Figure S4.** Luminescence production at room temperature with different expression levels of the three FARs . The S1 strain expressing Acr1, Aar or Ramo under an IPTG-inducible promoter were cultivated with varying concentrations of IPTG at room temperature (ca. 24°C) for 48 hours and the luminescence signals measured. The data is from the same experiment, but is presented in separate graphs for clarity. The control strains iluxAB (with wild type Acr1 expression) and S1 (FAR-negative strain) are shown in each graph as a reference. The averages of two replicates are shown.

**Analysis of WE composition by NMR**

Wax esters isolated by preparative TLC were analyzed by ^1^H NMR. Examples of ^1^H NMR spectra of a total lipid extract and a wax fraction isolated from it are given in Figures S5 and S6. For all the studied samples (W1 strain overexpressing either Acr1 or Ramo, and the control strain ADP1Δ3381) the ^1^H NMR spectra looked similar.

**Figure S5.** The ^1^H NMR spectrum of the total lipid extract from the biomass sample W1 + Acr1.

**Figure S6.** The ^1^H NMR spectrum of wax ester fraction isolated by preparative TLC from the total lipid extract of the biomass sample W1 + Acr1.

The average degree of unsaturation and the average length of fatty chains were calculated from NMR spectra as described earlier [1]. The analysis revealed no significant differences between the samples, and suggested an average structure of a WE molecule with 33-34 carbons and approximately one double bond.

**Analysis of WE composition by GC**

Wax esters isolated by preparative TLC were hydrolyzed and transmethylated using a modified procedure of Morrison and Smith [2]. The solution (14%) of BF_3_/methanol (Sigma-Aldrich Co.) was added to a dry sample of wax esters (1 mL/4-16 mg of a sample), containing methyl ester of fatty acid C_27:0_ as internal standard (1 mg/sample), in a sealed glass vial and the reaction mixture was heated at 100°C for 1 h. After cooling to room temperature an equal volume of water was added and the mixture was extracted with n-hexane (2 x 2 mL). Hexane phases were combined, the solvent was removed under a stream of nitrogen and a residue was dissolved in dichloromethane (1 mL) to obtain a sample for gas-chromatography (GC).

GC-analysis of fatty acid methyl esters (FAME) and fatty alcohols obtained after hydrolysis and transmethylation of wax fractions was carried out using Thermo Finnigan gas chromatograph equipped with a flame ionization detector. A 100% dimethylpolysiloxane column (Agilent Technologies) DB-1 (30m x 0.32 mm i.d., film thickness 0.25 µm) was used. Helium was the carrier gas at a flow rate of 1 mL/min; split flow was 50 mL/min; injection and detector temperature were 250°C. The oven temperature was programmed from 140°C for 5 min and then at the rate of 4°/min to 250°C and at 250°C with final hold for 10 min. The identification of major peaks was based on qualitative comparison with the C_8_-C_24_ FAME mix (Supelco^®^, Sigma-Aldrich, carbon chains C_8:0_, C_10:0_, C_12:0_, C_14:0_, C_16:1n-7_, C_16:0_, C_18:3n-3_, C_18:2n-6_, C_18:1n-9_, C_18:0_, C_20:0_, C_22:1n-9_, C_22:0_, C_24:0_) and the fatty alcohols (FAL) mixture (C_12_H_25_OH, C_14_H_29_OH, C_16_H_33_OH, C_18_H_37_OH) prepared in the lab.

The gas chromatogram of wax ester fractions is presented in Figure S7. Based on a comparison with the reference samples, the WEs are composed of fatty chains with 16 or 18 carbons and one or zero double bond. Taken together, both the GC and NMR methods suggest that the overexpression of Ramo or Acr1 does not significantly affect the chain length distribution or the degree of unsaturation of WEs and that WEs are predominantly composed of acyl chains with 16 or 18 carbons, with an average of one double bond per WE molecule.


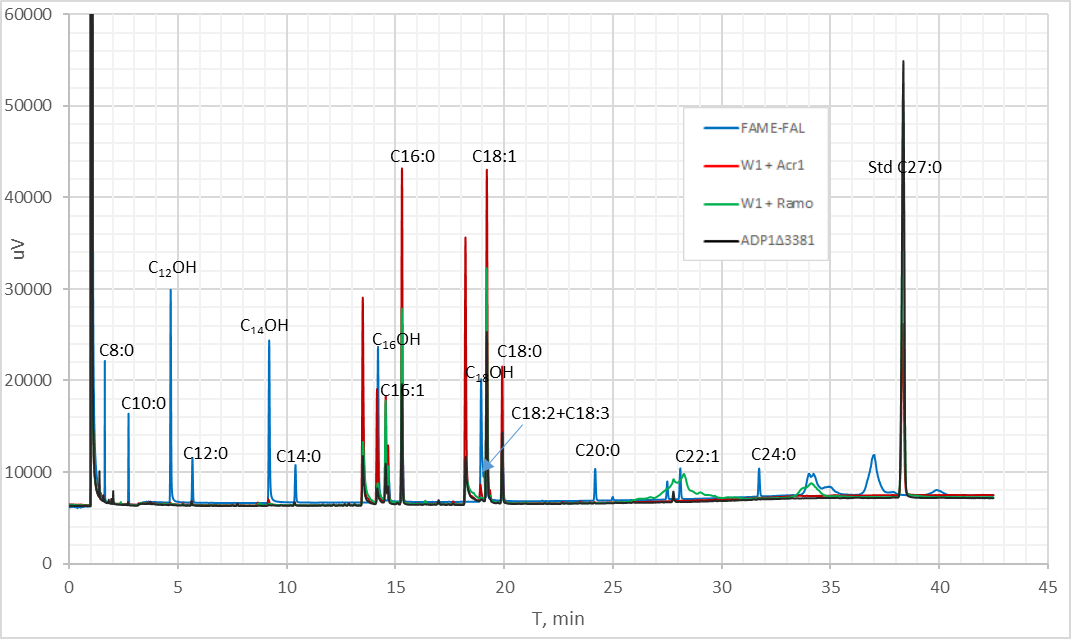


**Figure S7.** The gas chromatogram of purified, hydrolyzed and transmethylated samples. The control strain ADP1Δ3381 has the wild type Acr1 expression.

**Analysis of fatty alcohols by GC-MS**

**Table S1.** Fatty alcohol production of the FAR-overexpressing strains.

| Strain | CDW (g/L) | 1-Hexadecanol (mg/L) | 1-Octadecanol (mg/L) |
| --- | --- | --- | --- |
| Control | 8.7 ± 0.05 | 1.7 ± 0.4 | 0.59 ± 0.1 |
| W1 + Acr1 | 8.5 ± 0.1 | 8.1 ± 0.4 | 2.6 ± 0.2 |
| W1 + Aar | 8.1 ± 0.15 | 0.15 ± 0.02 | 0.15 ± 0.02 |

The W1 (ADP1Δ3381-3383::cm^r^) cells expressing either Acr1 or Aar under an IPTG-inducible promoter were cultivated at 30 °C for 48 hours, the lipids extracted and fatty alcohols measured with GC-MS. The average and standard deviation of two independent cultivations are shown. The control strain was ADP1Δ3381, which has the wild type Acr1 expression. CDW, cell dry weight.

**Table S2.** Primers used in the study.

| Name | Sequence 5' --> 3' | Description |
| --- | --- | --- |
| tl24 | CGTGGAATTGGTACCCTAGATGTTATTTCTTCAAAATTATGG | pIM1463 1/2 forward, KpnI |
| tl25 | GATGATCTTTCTCTAGAAGCGGCCGCGAATTCGCATGCTCTAGTAAGCTTCTCC | pIM1463 1/2 reverse, XbaI |
| tl26 | AGGGAGTCTAGAACTAGTAGCGGCCGCTGCAGAAAGGAGAAGCTTACTAGC | pIM1463 2/2 forward, XbaI |
| tl27 | TGCAGCGGCCCCCGAGGCCAAGCTTACCTGAAAGCCAAT | pIM1463 2/2 reverse, SfiI |
| tl17 | TGGAATTCGCGGCCGCTTCTAGAGAAAGAGGAGAAATACTAGATGATATCAATCAGGGAAAAACGCG | Acr1 forward, Biobrick |
| tl18 | GTTTCTTCCTGCAGCGGCCGCTACTAGTATTATTACCAGTGTTCGCCTGGG | Acr1 reverse, Biobrick |
| tl33 | CGGCCGCTTCTAGAGAAAGAGGAGAAATACTAGATGAATTACTTCGTCACCGG | RAMO, XbaI |
| tl34 | GGCCGCTACTAGTATTATTACCAATGCTCGCCCTTG | RAMO, SpeI |

**The nucleotide sequence of *acr* (ACIAD 3383):**

TTGATATCAATCAGGGAAAAACGCGTGAACAAAAAACTTGAAGCTCTCTTCCGAGAGAAT
GTAAAAGGTAAAGTGGCTTTGATCACTGGTGCATCTAGTGGAATCGGTTTGACGATTGCA
AAAAGAATTGCTGCGGCAGGTGCTCATGTATTATTGGTTGCCCGAACCCAAGAAACACTG
GAAGAAGTGAAAGCTGCAATTGAACAGCAAGGGGGACAGGCCTCTATTTTTCCTTGTGAC
CTGACTGACATGAATGCGATTGACCAGTTATCACAACAAATTATGGCCAGTGTCGATCAT
GTCGATTTCCTGATCAATAATGCAGGGCGTTCGATTCGCCGTGCCGTACACGAGTCGTTT
GATCGCTTCCATGATTTTGAACGCACCATGCAGCTGAATTACTTTGGTGCGGTACGTTTA
GTGTTAAATTTACTGCCACATATGATTAAGCGTAAAAATGGCCAGATCATCAATATCAGC
TCTATTGGTGTATTGGCCAATGCGACCCGTTTTTCTGCTTATGTCGCGTCTAAAGCTGCG
CTGGATGCCTTCAGTCGCTGTCTTTCAGCCGAGGTACTCAAGCATAAAATCTCAATTACC
TCGATTTATATGCCATTGGTGCGTACCCCAATGATCGCACCCACCAAAATTTATAAATAC
GTGCCCACGCTTTCCCCAGAAGAAGCCGCAGATCTCATTGTCTACGCCATTGTGAAACGT
CCAAAACGTATTGCGACGCACTTGGGTCGTCTGGCGTCAATTACCTATGCCATCGCACCA
GACATCAATAATATTCTGATGTCGATTGGATTTAACCTATTCCCAAGCTCAACGGCTGCA
CTGGGTGAACAGGAAAAATTGAATCTGCTACAACGTGCCTATGCCCGCTTGTTCCCAGGC
GAACACTGGTAA

**The nucleotide sequence of *ramo* (encoding for a putative short chain dehydrogenase WP_022976613.1):**

ATGAATTACTTCGTCACCGGCGCCACCGGTTTCATCGGCAAGCATCTGATCGAGCGCCTGCTGGCGCGCCCGGATGCCACCATCCACGTGCTGGTCCGCGCATCTTCGGAAGACAAGTTCGCCGCCTTGCAGGAGCGCTACGGTGATGCCGGCGACCGGCTGCAGATGGTCGCTGGCGACATCACCACGCCGGGCCTGGTGTCCGCCGCGGAGCTCAAGAAGCTGAAGGGCAAGGTCGGACACGTGTTTCACCTGGCCGCCGTCTACGACATGAACATGGACGATGCGACCGGCGATCGCATCAACAACGAAGGCACGCGCAACACCGTGGCCTTCGCCAACAGTCTGGGCGGCGACGTGGTGCTGCATCACGTGTCGAGCGTGGCGGTGGCCGGCGGCGATTTCGTCGGCACTTTCACCGAAGCGATGTTCGACGAAGGCCAGCCGGTCAAGCATCCGTACTTCCGGACCAAGTTCCAGTCCGAGAAGATCGTCCGTGACGAAGCCAAGGTGCCGTTCCGGGTCTATCGTCCCGGTGCTGTTGTCGGCCATTCGAAGACCGGCGAGATGGACAAGATCGACGGCCCTTACTACTTCTTCAAGACCATCCAGAAGCTCAGCCACCGGATTCCGAAGTGGCTGCCACTGCTCGGCATCGAAGGCGGCAAGGTGCCGATCGCGCCGGTCGACTACATCGCCGATGCGCTCGATGCGATCGCCCACAAGGACGGCCTGAACGGCCAGACCTTCCATCTGGTGCAGTCGAACAGCCCCAGCGTCGGCGACCTGATCCAGTCGATCCTGAAGGCCGCCCACGGGCCGCGCTTCAAGAAAAAGTTCGAGCTGCCGACGATGCCGGCCTCGATGCGCAAGTTCGGCGGCCAGATGGGCGGCGCCTTGCCGGCCAGCGTCAAGAAGCAGATCGCCAAGGCGATCGGCGCACCGCTGTCGGTGCTCGGCTACATCACCAACCGTGCCGTGTTCGACGACAAGAACGCCCGCGCCGCGCTCAAGGGCACCGGCATCAAGTGCCCGGAATTCCGCGAGTACGCGAAGCACCTGTGGTCGTACTGGGAGCAGTTCCTCGACATCCACTACGAGCCGAGTGCCGAGCTGATCGCCAAGGTGAAGGGCAAGGTGATCCTGGTCACCGGCGCCTCATCGGGCATCGGCTTCACCACCGCGAAGAACCTGGCGATTGCCGGCGCCCGGGTCATCCTGGTGGCGCGTACCGAATCGAACCTGATCGAAACTCAGGAGATCATTTCCCGGGCCGGCGGCGAGAGCTATGTCTACCCCTGCGATCTGATCGACATGAAGGCGATCGACGCCATGGCCGCGAAAGTGCTGCGCGACTTCGGCCACGTCGACATCCTGATCAACAACGCCGGCCGCTCGATCCGCCGCGCAGTGATGGAAAGCTTCGACCGCTTCCATGACTTCGAGCGGACCATGGAGCTGAACTACTACGGCGCCGTGCGCCTGATCATGGCGCTGCTGCCGACGATGACCGCGCGCAAGAACGGCCACATCATCAACATCAGCTCGATCGGCGTGCTGGCCAACGCGGCACGCTTCTCGGCCTATGTCGCCTCCAAGGCCGCGCTCGATGCCTTCACCCGCTGCCTGTCGGCCGAAGTGAAAGGCAGCAACATCCGCACCACGGTGATCTACATGCCGCTGGTGCGCACGCCGATGATCGCTCCGACCAAGATCTACAGCTACGTGCCGACCTGGTCCCCGGACGACGCCGCCGACACCGTGATCAAGGCGATCCTCGACGAACCGAAATCGATCGCGACAACTCTGGGTACTGCCGCTGCGGTCAGCTATGCGATCTGGCCGAAGGTCAACGACTACATCCTGTCGAAAGGCTTCCAGCTGTTCCCGTCATCAACCGCCGCACGTGGCTCGAAGGACAAGGACTCGAAGGCCGACAAGCCGACCCTCGAACAGGTGGTGTTCGCGAATGTGTTCAAGGGCGAGCATTGGTAA

**The nucleotide sequence of *aar* (*S. elongatus PCC7942_orf 1594),* codon-optimized and including the restriction sites and the RBS*:***

GAATTCGCGGCCGCTTCTAGAGAAAGAGGAGAAATACTAGATGTTTGGTTTAATTGGCCATTTGACCTCGCTGGAACAGGCACGTGATGTTAGTCGTCGCATGGGCTATGATGAATATGCGGATCAGGGTCTGGAATTTTGGAGTTCTGCCCCGCCACAAATTGTGGATGAAATCACCGTTACATCTGCTACAGGCAAAGTGATTCATGGTCGTTATATCGAAAGCTGTTTTCTGCCGGAAATGCTGGCAGCGCGTCGCTTTAAAACTGCCACGCGCAAAGTTTTAAATGCTATGAGTCATGCACAGAAACATGGTATTGATATCAGCGCGCTGGGTGGCTTTACCTCAATCATCTTTGAAAACTTTGATTTAGCCTCTTTGCGTCAAGTACGCGATACCACACTGGAATTTGAACGTTTTACTACGGGCAACACCCATACAGCGTATGTAATTTGTCGCCAGGTCGAAGCCGCTGCAAAAACTCTGGGTATTGATATCACTCAAGCGACGGTTGCCGTGGTTGGTGCTACGGGCGATATTGGTTCGGCAGTATGCCGTTGGTTGGATCTGAAATTAGGTGTCGGCGATTTGATTCTGACTGCTCGTAATCAGGAACGCCTGGATAACTTACAAGCAGAACTGGGTCGTGGCAAAATTTTACCTTTGGAAGCGGCCTTACCGGAAGCGGATTTTATTGTATGGGTCGCCTCAATGCCGCAGGGTGTAGTCATCGATCCAGCAACATTAAAACAACCTTGCGTGTTGATTGATGGTGGCTATCCAAAAAATTTGGGTAGCAAAGTGCAAGGTGAAGGCATTTATGTTCTGAACGGTGGCGTGGTTGAACATTGTTTTGATATTGATTGGCAGATCATGAGTGCAGCAGAAATGGCACGTCCTGAACGCCAAATGTTTGCATGCTTTGCGGAAGCCATGCTGTTAGAATTTGAAGGCTGGCATACCAATTTTTCATGGGGTCGTAACCAGATTACAATCGAAAAAATGGAAGCGATCGGCGAAGCCTCGGTGCGTCATGGTTTTCAACCGCTGGCTTTAGCAATTTAATAATACTAGTAGCGGCCGCTGCAG


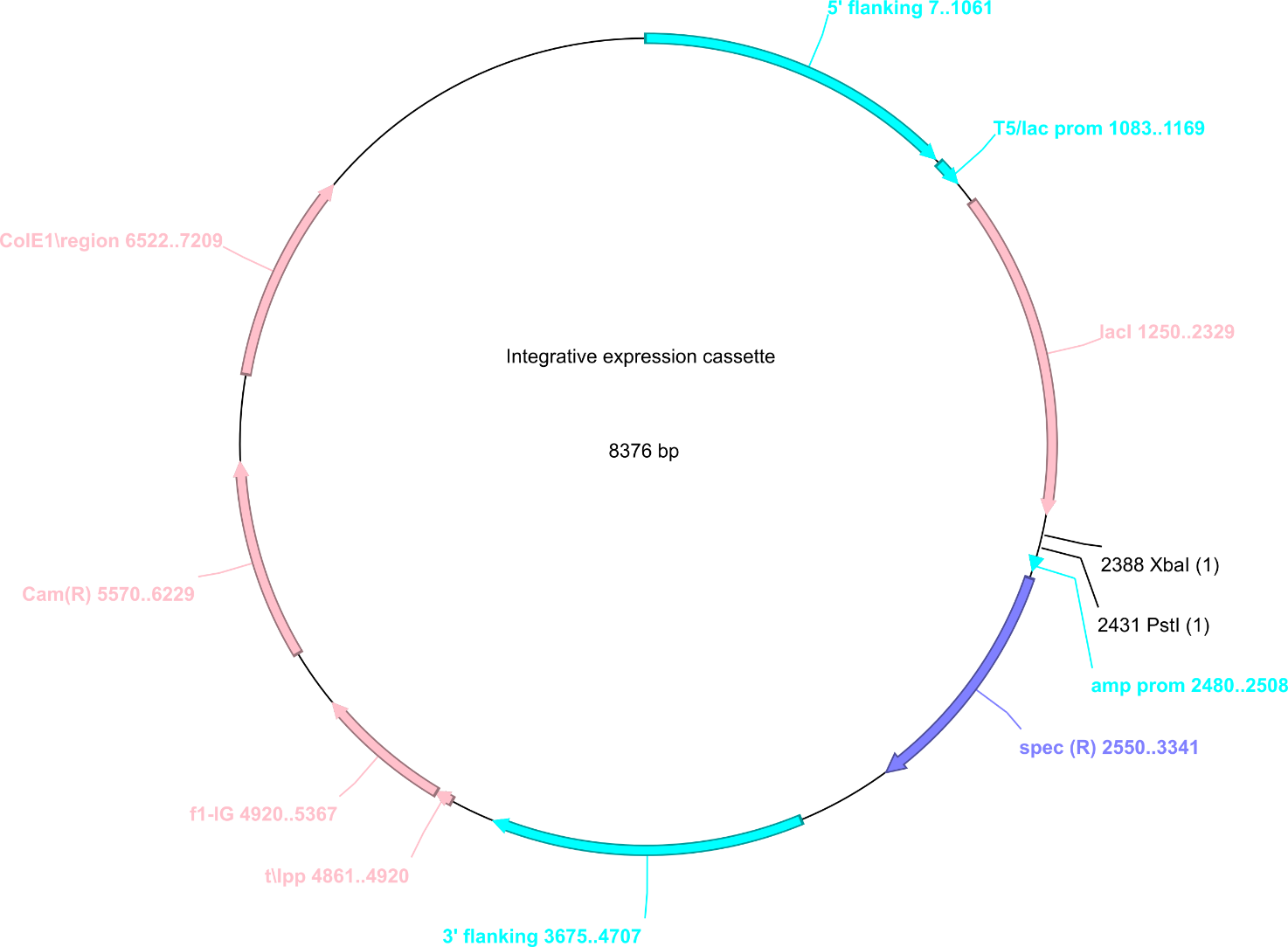


**Figure S8.** The integrative expression cassette. The integrative cassette used for the FAR overexpression includes the *lacI* gene and the cloning site with XbaI and PstI restriction sites under the T5/lac promoter, as well as the spectinomycin resistance marker under its own promoter. The FAR genes were cloned to this plasmid using XbaI and PstI. The 5’ and 3’ flanking sites facilitate the homologous recombination of the integrative part of the plasmid to the *A. baylyi* genome. Other relevant components of the plasmid backbone are the ColE1 origin of replication as well as the chloramphenicol resistance marker in the non-integrative part of the plasmid.

**The nucleotide sequence of the integrative expression cassette:**

GGTACCCTAGATGTTATTTCTTCAAAATTATGGATTAATTTAAATTGTTGAGCCGACATTTTATTACCCTCTTATCAAACCGTACCTTTCACATAACGAATGAATGAATACCGTACATGGAGTGCGGCCAACCCACAGCGAACATCATATTTCGCATCCATCACCGTACGGTTTTCCGTTTTAAGCTCTGCCCATGATCTATCATGGAAATAACGGCTAATGATCACCTGCATCCACTCAAGTGTCGTTTCACTGTCTGTACCATTAATAATATCCAGTACTAAACGTTGTACGGCACGAGCTTCATTATCGTTAATCTGACACGACACTTTGTGACGTATAGCTTGTTGTACTCCTTGAGCATCACAAAGGTAATAAGCAATAAGTTTAGCTCGATCTTTCTTCTTTACACGTACCTTGGCTTTCTTCATTGCAATAGCAATCGGGCTATCGGAATACTGTCCACCACGACAAGAACGTTGCCACGCTCCACATTGGCGTAACCAATCTGGCAAATCATACTTGCTCCAATCCACCGTCTGCATAATGTGCACTGCTGTATTCATCTCATCACCTAATTTGTTTCAAGTTAAATTTTATAAGCGTTATTGTTTTATGGTTCTGCCTGCTCCTCTACCGATCTAAAACGACAAGTTTCGAGATAATCCAGTACTCGAACTGCACCGCGTTTACCGTGTCGGTTTTTCACTACAATCAGCTCTGTGATTCCCATCGGTTTGGTTGAGTCTTCTGGATCGGTTAGGGGGTTAACAAGGATGATCTGGTCTGCATCTTGCTCGATTTGTCCAGATTCTTTGATATCTGATGCTTTAGGACGTTTGCCCTTCTCTGCCTCACGGTTGAGCTGTACCAGTGCAATGACAGGACATTCAAACTCTTTCGCCATGGATTTTAATTCACGGCTGATGGAACTGACTTCCTGAAAGCGATCTTTCTTGCTCGGGTCTCTGAGCAGTTGTAAATAATCCACGATGATGCAGCCCAATTCCTTGTAACGGCGTTTGGCTCGACGTGCATAGGAACGGACCTCACTCAAGCTTACTAGAACTATAGCTAGTCGAGAAATCATAAAAAATTTATTTGCTTTGTGAGCGGATAACAATTATAATAGATTCAATTGTGAGCGGATAACAATTTCACACAGAATTAATTCATTAAAGAGGAGAAATTAACCATGGGCAGCAGCCATCACCATCATCACCACAGCCAGGATCCGGCCCTGAGGGCCGTGAAACCAGTAACGTTATACGATGTCGCAGAGTATGCCGGTGTCTCTTATCAGACCGTTTCCCGCGTGGTGAACCAGGCCAGCCACGTTTCTGCGAAAACGCGGGAAAAAGTGGAAGCGGCGATGGCGGAGCTGAATTACATTCCCAACCGCGTGGCACAACAACTGGCGGGCAAACAGTCGTTGCTGATTGGCGTTGCCACCTCCAGTCTGGCCCTGCACGCGCCGTCGCAAATTGTCGCGGCGATTAAATCTCGCGCCGATCAACTGGGTGCCAGCGTGGTGGTGTCGATGGTAGAACGAAGCGGCGTCGAAGCCTGTAAAGCGGCGGTGCACAATCTTCTCGCGCAACGCGTCAGTGGGCTGATCATTAACTATCCGCTGGATGACCAGGATGCCATTGCTGTGGAAGCTGCCTGCACTAATGTTCCGGCGTTATTTCTTGATGTCTCTGACCAGACACCCATCAACAGTATTATTTTCTCCCATGAAGACGGTACGCGACTGGGCGTGGAGCATCTGGTCGCATTGGGTCACCAGCAAATCGCGCTGTTAGCGGGCCCATTAAGTTCTGTCTCGGCGCGTCTGCGTCTGGCTGGCTGGCATAAATATCTCACTCGCAATCAAATTCAGCCGATAGCGGAACGGGAAGGCGACTGGAGTGCCATGTCCGGTTTTCAACAAACCATGCAAATGCTGAATGAGGGCATCGTTCCCACTGCGATGCTGGTTGCCAACGATCAGATGGCGCTGGGCGCAATGCGCGCCATTACCGAGTCCGGGCTGCGCGTTGGTGCGGATATCTCGGTAGTGGGATACGACGATACCGAAGACAGCTCATGTTATATCCCGCCGTTAACCACCATCAAACAGGATTTTCGCCTGCTGGGGCAAACCAGCGTGGACCGCTTGCTGCAACTCTCTCAGGGCCAGGCGGTGAAGGGCAATCAGCTGTTGCCCGTCTCACTGGTGAAAAGAAAAACCACCCTGGCGCCCAATACGCAAACCGCCTCTCCCCGCGCGTTGGCCGATTCATTAATGCAGCTGGCACGACAGGTTTCCCGACTGGAAAGCGGGCAGGGCCTATGCGGCCGGTAGAAAGGAGAAGCTTACTAGAGCATGCGAATTCGCGGCCGCTTCTAGAGAAAGAGGAGAAATACTAGTAATACTAGTAGCGGCCGCTGCAGAAAGGAGAAGCTTACTAGCTATTTGTTTATTTTTCTAAATACATTCAAATATGTATCCGCTCATGAGACAATAACCCTGATAAATGCTTCAATAATATTGAAAAAGGAAGAGTATGAGGGAAGCGGTGATCGCCGAAGTATCGACTCAACTATCAGAGGTAGTTGGCGTCATCGAGCGCCATCTCGAACCGACGTTGCTGGCCGTACATTTGTACGGCTCCGCAGTGGATGGCGGCCTGAAGCCACACAGTGATATTGATTTGCTGGTTACGGTGACCGTAAGGCTTGATGAAACAACGCGGCGAGCTTTGATCAACGACCTTTTGGAAACTTCGGCTTCCCCTGGAGAGAGCGAGATTCTCCGCGCTGTAGAAGTCACCATTGTTGTGCACGACGACATCATTCCGTGGCGTTATCCAGCTAAGCGCGAACTGCAATTTGGAGAATGGCAGCGCAATGACATTCTTGCAGGTATCTTCGAGCCAGCCACGATCGACATTGATCTGGCTATCTTGCTGACAAAAGCAAGAGAACATAGCGTTGCCTTGGTAGGTCCAGCGGCGGAGGAACTCTTTGATCCGGTTCCTGAACAGGATCTATTTGAGGCGCTAAATGAAACCTTAACGCTATGGAACTCGCCGCCCGACTGGGCTGGCGATGAGCGAAATGTAGTGCTTACGTTGTCCCGCATTTGGTACAGCGCAGTAACCGGCAAAATCGCGCCGAAGGATGTCGCTGCCGACTGGGCAATGGAGCGCCTGCCGGCCCAGTATCAGCCCGTCATACTTGAAGCTAGACAGGCTTATCTTGGACAAGAAGAAGATCGCTTGGCCTCGCGCGCAGATCAGTTGGAAGAATTTGTCCACTACGTGAAAGGCGAGATCACCAAGGTAGTCGGCAAATAATGTCTAACAATTCGTTCAAGCCGAGGGGCCGCAAGATCCGGCCACGATGACCCGGTCGTCGGTTCAGGGCAGGGTCGTTAAATAGCCGCTTATGTCTATTGCTGGTTTACCGGTTTATTGACTACCGGAAGCAGTGTGACCGTGTGCTTCTCAAATGCCTGAGGTTTCAGCAAAAAACCCCTCAAGACCCGTTTAGAGGCCCCAAGGGGTTATGCTAGTTATTGCTCAGCGGTGGCAGCAGCCTAGGTTAATTAAGCTGCGCTAGTAGACGAGTCCATGTGCTGGCGTTCAAATTTCGCAGCAGCGGTTTCTTTACCAGACTCGACAAGCTTACTAGAGTGTTCATATTGACCTCGCTTAGTGTGGTTAATACGCCGCTTCTTGTTACTGCAAGAGGCGGTTTTTTTATGGGTGTACACATGACTGCACCTGTTGATTCAGTTCAGATAGTGCTTTGTGCATCTCATGGATGACTCTGGCCATATCCAGTGCTTCGCCTTGGGTAATTCGCCCATCGGCCATCATTTCTTTAAACAGTGCTGATATATCGCCCTTCTTGATGCCTATGCATAGGAAGGTATCCATCAGACTGGTATCTCGCTGGCTCTCGGGTATGTCTGGCAGGTCAATTGCCACCTTTCCGAGTCGTGCACACATTTCCTGCAATATCCGATAGTCCCCTGTAATCTCCATCAGCTTGACTGCCTCGAGCAATGTAATGTGATGGGTATGTGTGTTTGGGTTGACCTTGCTATTGAGCACCGCAGGGCTTTTGATGCCTAAACGTGATGCAAGTGCAGATGCACCACCCAGAAAGTCGTGAACGGTGTGATAGGCAGCATCTAATATGTTCATGGCGGGTTCCTTTGAACGTGTTTATTAGATGGGTGCTGACATAAGATTGGATTTATGGTTAGGACGTAATTCAATCCAAATATCTTCGTAATCATCTGGAAATAAATCTTTGCGAGTGCATAGACCTTTATCTTCAGCAATTACAGCTAAGCGGATTTTGCGATCTCTGGGAATTGCTTTCCATCCACTTACGGATGCAGCGGTGATACCTAAAAATCTAGCAACAGCAGTTACACCACCTAAAAGCTCAATAAATTGATCATCAGTCATGTTGATCTCCTAATTTTATTGCCTCAATTATTAGGTATTCCTTATATTTTATCAATAGGAATACCTTATTTATTTTATGTTAGGATTTCCTAATAGACTAGGTAAGATCATGAAAACATTAGCTGAACGACTTAAATATGCGATGGAAATTTTGCCACCTAAGAAAATCAAGGGTGTCGAACTTGCTCGTGTAGTTGGAGTTAAACCACCATCTGTCAGCGATTGGCTTTCAGGTAAGCTTGGCCTCGGGGGCCGAATTCGGAGGCGGTGGCTCTATGAGTGTTTTAGTGTATTCTTTCGCCTCTTTCGTTTTAGGTTGGTGCCTTCGTAGTGGCATTACGTATTTTACCCGTTTAATGGAAACTTCCTCATGATAAGCTTGACCTGTGAAGTGAAAAATGGCGCACATTGTGCGACATTTTTTTTGTCTGCCGTTTACCGCTACTGCGTCACGGATCCCCACGCGCCCTGTAGCGGCGCATTAAGCGCGGCGGGTGTGGTGGTTACGCGCAGCGTGACCGCTACACTTGCCAGCGCCCTAGCGCCCGCTCCTTTCGCTTTCTTCCCTTCCTTTCTCGCCACGTTCGCCGGCTTTCCCCGTCAAGCTCTAAATCGGGGCATCCCTTTAGGGTTCCGATTTAGTGCTTTACGGCACCTCGACCCCAAAAAACTTGATTAGGGTGATGGTTCACGTAGTGGGCCATCGCCCTGATAGACGGTTTTTCGCCCTTTGACGTTGGAGTCCACGTTCTTTAATAGTGGACTCTTGTTCCAAACTGGAACAACACTCAACCCTATCTCGGTCTATTCTTTTGATTTATAAGGGATTTTGCCGATTTCGGCCTATTGGTTAAAAAATGAGCTGATTTAACAAAAATTTAACGCGAATTTTAACAAAATATTAACGTTTACAATTTCAGGTGGCACTTTTCGGGGAAATGTGCGCGGAACCCCTATTTGTTTATTTTTCTAAATACATTCAAATATGTATCCGCTCATGTCGAGACGTTGGGTGAGGTTCCAACTTTCACCATAATGAAATAAGATCACTACCGGGCGTATTTTTTGAGTTATCGAGATTTTCAGGAGCTAAGGAAGCTAAAATGGAGAAAAAAATCACTGGATATACCACCGTTGATATATCCCAATGGCATCGTAAAGAACATTTTGAGGCATTTCAGTCAGTTGCTCAATGTACCTATAACCAGACCGTTCAGCTGGATATTACGGCCTTTTTAAAGACCGTAAAGAAAAATAAGCACAAGTTTTATCCGGCCTTTATTCACATTCTTGCCCGCCTGATGAATGCTCATCCGGAGTTCCGTATGGCAATGAAAGACGGTGAGCTGGTGATATGGGATAGTGTTCACCCTTGTTACACCGTTTTCCATGAGCAAACTGAAACGTTTTCATCGCTCTGGAGTGAATACCACGACGATTTCCGGCAGTTTCTACACATATATTCGCAAGATGTGGCGTGTTACGGTGAAAACCTGGCCTATTTCCCTAAAGGGTTTATTGAGAATATGTTTTTCGTCTCAGCCAATCCCTGGGTGAGTTTCACCAGTTTTGATTTAAACGTGGCCAATATGGACAACTTCTTCGCCCCCGTTTTCACCATGGGCAAATATTATACGCAAGGCGACAAGGTGCTGATGCCGCTGGCGATTCAGGTTCATCATGCCGTCTGTGATGGCTTCCATGTCGGCAGAATGCTTAATGAATTACAACAGTACTGCGATGAGTGGCAGGGCGGGGCGTAATTTTTTTAAGGCAGTTATTGGTGCCCTTAAACGCCTGGTGCTACGCCTGAATAAGTGATAATAAGCGGATGAATGGCAGAAATTCGAAAGCAAATTCGACCCGGTCGTCGGTTCAGGGCAGGGTCGTTAAATAGCCGCTTATGTCTATTGCTGGTTTACCGGTTTATTGACTACCGGAAGCAGTGTGACCGTGTGCTTCTCAAATGCCTGAGGCCAGTTTGCTCAGGCTCTCCCCGTGGAGGTAATAATTGCTCGACATGACCAAAATCCCTTAACGTGAGTTTTCGTTCCACTGAGCGTCAGACCCCGTAGAAAAGATCAAAGGATCTTCTTGAGATCCTTTTTTTCTGCGCGTAATCTGCTGCTTGCAAACAAAAAAACCACCGCTACCAGCGGTGGTTTGTTTGCCGGATCAAGAGCTACCAACTCTTTTTCCGAAGGTAACTGGCTTCAGCAGAGCGCAGATACCAAATACTGTCCTTCTAGTGTAGCCGTAGTTAGGCCACCACTTCAAGAACTCTGTAGCACCGCCTACATACCTCGCTCTGCTAATCCTGTTACCAGTGGCTGCTGCCAGTGGCGATAAGTCGTGTCTTACCGGGTTGGACTCAAGACGATAGTTACCGGATAAGGCGCAGCGGTCGGGCTGAACGGGGGGTTCGTGCACACAGCCCAGCTTGGAGCGAACGACCTACACCGAACTGAGATACCTACAGCGTGAGCTATGAGAAAGCGCCACGCTTCCCGAAGGGAGAAAGGCGGACAGGTATCCGGTAAGCGGCAGGGTCGGAACAGGAGAGCGCACGAGGGAGCTTCCAGGGGGAAACGCCTGGTATCTTTATAGTCCTGTCGGGTTTCGCCACCTCTGACTTGAGCGTCGATTTTTGTGATGCTCGTCAGGGGGGCGGAGCCTATGGAAAAACGCCAGCAACGCGGCCTTTTTACGGTTCCTGGCCTTTTGCTGGCCTTTTGCTCACATGACCCGACACCATCGAATGGCGCAAAACCTTTCGCGGTATGGCATGATAGCGCCCGGAAGAGAGTCAATTCAGGGTGGTGAATGTGAAACCAGTAACGTTATACGATGTCGCAGAGTATGCCGGTGTCTCTTATCAGACCGTTTCCCGCGTGGTGAACCAGGCCAGCCACGTTTCTGCGAAAACGCGGGAAAAAGTGGAAGCGGCGATGGCGGAGCTGAATTACATTCCCAACCGCGTGGCACAACAACTGGCGGGCAAACAGTCGTTGCTGATTGGCGTTGCCACCTCCAGTCTGGCCCTGCACGCGCCGTCGCAAATTGTCGCGGCGATTAAATCTCGCGCCGATCAACTGGGTGCCAGCGTGGTGGTGTCGATGGTAGAACGAAGCGGCGTCGAAGCCTGTAAAGCGGCGGTGCACAATCTTCTCGCGCAACGCGTCAGTGGGCTGATCATTAACTATCCGCTGGATGACCAGGATGCCATTGCTGTGGAAGCTGCCTGCACTAATGTTCCGGCGTTATTTCTTGATGTCTCTGACCAGACACCCATCAACAGTATTATTTTCTCCCATGAAGACGGTACGCGACTGGGCGTGGAGCATCTGGTCGCATTGGGTCACCAGCAAATCGCGCTGTTAGCGGGCCCATTAAGTTCTGTCTCGGCGCGTCTGCGTCTGGCTGGCTGGCATAAATATCTCACTCGCAATCAAATTCAGCCGATAGCGGAACGGGAAGGCGACTGGAGTGCCATGTCCGGTTTTCAACAAACCATGCAAATGCTGAATGAGGGCATCGTTCCCACTGCGATGCTGGTTGCCAACGATCAGATGGCGCTGGGCGCAATGCGCGCCATTACCGAGTCCGGGCTGCGCGTTGGTGCGGATATCTCGGTAGTGGGATACGACGATACCGAAGACAGCTCATGTTATATCCCGCCGTCAACCACCATCAAACAGGATTTTCGCCTGCTGGGGCAAACCAGCGTGGACCGCTTGCTGCAACTCTCTCAGGGCCAGGCGGTGAAGGGCAATCAGCTGTTGCCCGTCTCACTGGTGAAAAGAAAAACCACCCTGGCGCCCAATACGCAAACCGCCTCTCCCCGCGCGTTGGCCGATTCATTAATGCAGCTGGCACGACAGGTTTCCCGACTGGAAAGCGGGCAGTGAGC

**Additional References**

1. Efimova E, Marjakangas JM, Lakaniemi AM, Koskinen PE, Puhakka JA: **Lipid profile characterization of wastewaters from different origins.** Water Sci Technol 2013, **68**(11):2505-2514.

2. MORRISON WR, SMITH LM: **Preparation of Fatty Acid Methyl Esters and Dimethylacetals from Lipids with Boron Fluoride--Methanol.** J Lipid Res 1964, **5**:600-608.
